# Medium-Dose Compared With High-Dose Inhaled Corticosteroids in Combination Triple Inhaler Therapy for Asthma: A Systematic Review and Meta-Analysis

**DOI:** 10.1016/j.chpulm.2026.100240

**Published:** 2026-02-21

**Authors:** Samantha Warhurst, Ryan Cullen, Ross Sayers, Francesca Lynch, Joseph Kulathinal, Mark Weatherall, Richard W. Beasley, Jonathan H. Noble

**Affiliations:** a Medical Research Institute of New Zealand, Wellington, New Zealand; b University of Otago, Wellington, New Zealand; c Victoria University of Wellington, Wellington, New Zealand

**Keywords:** asthma, combination triple inhaler therapy, corticosteroid stewardship, dose response, inhaled corticosteroids

## Abstract

**Background:**

Combination inhaled corticosteroid (ICS)/long-acting beta_2_-agonist (LABA)/long-acting muscarinic antagonist (LAMA) triple therapy is used in severe asthma. The clinical effect of high-dose (HD) vs medium-dose (MD) ICSs in ICS/LABA/LAMA is unclear.

**Research Question:**

What is the clinical effect of HD compared with MD ICSs in ICS/LABA/LAMA treatment of asthma?

**Study Design and Methods:**

A systematic review and meta-analysis of randomized controlled trials including > 1 ICS dose in ICS/LABA/LAMA inhalers compared the outcomes of HD and MD ICS/LABA/LAMA. The primary outcome was the proportion of participants with ≥ 1 severe asthma exacerbations. Secondary outcomes included FEV_1_, the Asthma Control Questionnaire, emergency department visits, and hospital admissions. Certainty of evidence was assessed using the Grading of Recommendations, Assessment, Development and Evaluations domains.

**Results:**

Three randomized controlled trials (N = 3,804) were identified, comparing MD and HD ICS/LABA/LAMA containing fluticasone or mometasone. When comparing HD with MD ICS/LABA/LAMA for at least 1 severe exacerbation, the Peto OR was 0.81 (95% CI, 0.68-0.96; *P* = .01). This corresponds to a number needed to treat to prevent 1 severe exacerbation of 32.9. HD ICS/LABA/LAMA led to a higher FEV_1_ (mean difference, 50.8 mL; 95% CI, 29.5-72.2 mL; *P* < .001) and a lower (improvement) Asthma Control Questionnaire (mean difference, −0.09; 95% CI, −0.15 to −0.04; *P* < .001) than MD ICS/LABA/LAMA. There was no statistically significant difference in emergency department visits (Peto OR, 0.51; 95% CI, 0.25-1.04; *P* = .07) or hospitalizations (Peto OR, 0.65; 95% CI, 0.33-1.29; *P* = .22) between HD and MD ICS/LABA/LAMA.

**Interpretation:**

Our results show that HD compared with MD ICS/LABA/LAMA reduces the odds of a severe exacerbation by about 20%. This absolute risk reduction was modest but may be clinically relevant for patients with high baseline exacerbation rates and those with high type 2 inflammation. At both the individual and population level, the use of HD ICS/LABA/LAMA needs to be considered alongside the potential systemic side effects.

**Clinical Trial Registration:**

PROSPERO Registry; No.: CRD42025634340; URL: https://www.crd.york.ac.uk/prospero/

**Take-Home Points**

**Study** **Question:** What is the magnitude of the benefit of high-dose vs medium-dose inhaled corticosteroids when delivered in inhaled corticosteroid/long-acting beta-agonist/long-acting antimuscarinic therapy for asthma?

**Results:** High-dose compared with medium-dose inhaled corticosteroids in relation to triple asthma therapy were associated with a statistically significant odds ratio for severe exacerbations of about 0.8.

**Interpretation:** High-dose compared with medium dose inhaled corticosteroids were associated with a modest absolute risk reduction in severe exacerbations that might be of most relevance to those at high risk, such as those with high baseline exacerbation rates or high type 2 inflammation, but should be weighed against the risk of adverse effects.

Inhaled corticosteroids (ICSs) are highly effective antiinflammatory medications for asthma^1^ and are frequently used in fixed-dose combination with inhaled long-acting beta_2_-agonists (LABAs) in adolescent and adult asthma.^1^ For patients with asthma not controlled with maintenance medium-dose (MD) or high-dose (HD) ICS/LABA, add-on therapy with long-acting muscarinic antagonists (LAMAs) may be considered. Combination ICS/LABA/LAMA triple inhalers are now widely available and licensed for use in asthma.

Studies investigating the clinical dose-response of ICSs, delivered without combination LABAs, report minimal additional clinical efficacy of ICSs above low to medium doses.^2^, ^3^, ^4^ We recently reported that for ICSs delivered as maintenance therapy in combination ICS/LABA inhalers, HD ICS/LABA results in an odds ratio of a severe asthma exacerbation compared with MD ICS/LABA of 0.81, with no evidence for a clinically meaningful effect on lung function, asthma symptoms, or hospitalizations due to asthma.^5^

The magnitude of the additional benefit of HD vs MD ICSs when delivered in ICS/LABA/LAMA inhaler therapy is uncertain. The aim of this systematic review and meta-analysis is to investigate the clinical dose-response relationship for different maintenance ICS doses when given in combination ICS/LABA/LAMA inhaler formulations.

## Study Design and Methods

A prospectively registered systematic review and meta-analysis (PROSPERO Registration No. CRD42025634340) was performed, and is reported, according to the Preferred Reporting Items for Systematic Reviews and Meta-Analyses statement.^6^

### Search Strategy and Eligibility Criteria

A systematic search of MEDLINE, Embase, Cochrane Register of Controlled Trials, and Clinical Trials.gov was last conducted on December 31, 2024, before data extraction. The complete search terms are described in supplementary materials e-Appendix 1. In addition, reference lists from included studies were reviewed. Eligible studies were randomized controlled trials (RCTs) ≥ 12 weeks in duration that included patients with asthma ≥ 12 years of age who were randomly allocated to at least 2 different Global Initiative for Asthma (GINA) dose categories of ICSs in a single fixed-combination ICS/LABA/LAMA inhaler. The ICS/LABA/LAMA must have been delivered by the same inhaler device using the same specific ICSs, and with the same daily dose of LABA and LAMA. RCTs that allocated study participants to different ICS doses based on prestudy ICS doses were excluded. In addition, RCTs of asthma biologic therapies and antiinflammatory ICS/formoterol reliever therapies were excluded, and those that included participants with COPD or asthma-COPD overlap syndrome.

### Selection and Data Collection Process

Two reviewers independently screened study titles, abstracts, and full texts. Two reviewers independently extracted the study data. This process was conducted on Covidence systematic review software (Veritas Health Innovation). Clinical trial registry documentation and pharmaceutical clinical trial repositories were reviewed for outcome data for all included studies, where available. Disagreements were resolved by consensus or with a third reviewer. Study representatives were contacted if the primary outcome data were missing, and additional outcome data for 1 study^7^ were identified from this.

### Study Data and Outcomes

Study participant and intervention characteristics, eligibility criteria, and sponsor details were extracted in addition to study outcomes. The primary outcome was the proportion of participants with ≥ 1 severe asthma exacerbations, where a severe asthma exacerbation was defined as a worsening of asthma requiring treatment with oral corticosteroids. Secondary outcomes were change from baseline in a patient-reported outcome measure of asthma control, either the Asthma Control Questionnaire (ACQ) or Asthma Control Test, change from baseline in prebronchodilator or trough FEV_1_, the proportion of participants with ≥ 1 emergency department (ED) attendances for asthma, the proportion of participants with ≥ 1 hospital admissions for asthma, the proportion of participants with ≥ 1 serious adverse events, deaths related to asthma, and all-cause deaths.

### Risk of Bias Assessment and Certainty of Evidence

Two reviewers independently assessed each study and outcome using the Cochrane risk of bias in randomized trials^8^ and the certainty of evidence using Grading of Recommendations, Assessment, Development and Evaluations (GRADE) domains.^9^ The final risk of bias and GRADE ratings were determined by the consensus of the reviewers.

### Data Synthesis

Outcomes from included studies were pooled into their respective GINA ICS category based on the GINA 2024 report^10^: low-dose (LD) ICS/LABA/LAMA, MD ICS/LABA/LAMA, and HD ICS/LABA/LAMA. For this review, fluticasone furoate 100 μg was considered MD as listed in a previous meta-analysis, and based on the pharmaceutical datasheet where fluticasone furoate 100 μg is reported to be equivalent to fluticasone propionate 500 μg.^11^^,^^12^ When combined with indacaterol and glycopyrronium in a dry powder inhaler, mometasone furoate 80 μg was considered MD and mometasone furoate 160 μg was considered HD.^13^

In the event no studies of LD ICS/LABA/LAMA were identified, and so pairwise comparisons between HD and MD ICS/LABA/LAMA were made. The prespecified analysis for the primary outcome, the proportion of participants with ≥ 1 severe asthma exacerbations, was with Peto OR because we were uncertain if the data would be sparse and this is the preferred technique with sparse outcome data. A prespecified secondary analysis of the primary outcome was to estimate the absolute risk difference and related number needed to treat to prevent 1 severe exacerbation.

The secondary continuous outcome, change from baseline FEV_1_, was pooled using inverse variance-weighted meta-analysis on the scale of measurement, based on reported means, SDs, and participant counts. For change from baseline patient-reported outcome measure, it was planned to use inverse variance weighting of standardized effect sizes with small sample correction. In the event the ACQ-7 was the only patient-reported outcome measure assessed, the same approach was used as for FEV_1_. The remaining secondary outcomes are categorical variables and Peto OR was used. Homogeneity statistics were calculated for each analysis and an estimate of the I^2^ statistics. Although in the event few studies were identified, a formal test of publication bias used a correlation coefficient for effect size in relation to variance.

Meta-analyses were performed using SAS (version 9.4; SAS Institute).

## Results

**Figure 1:**
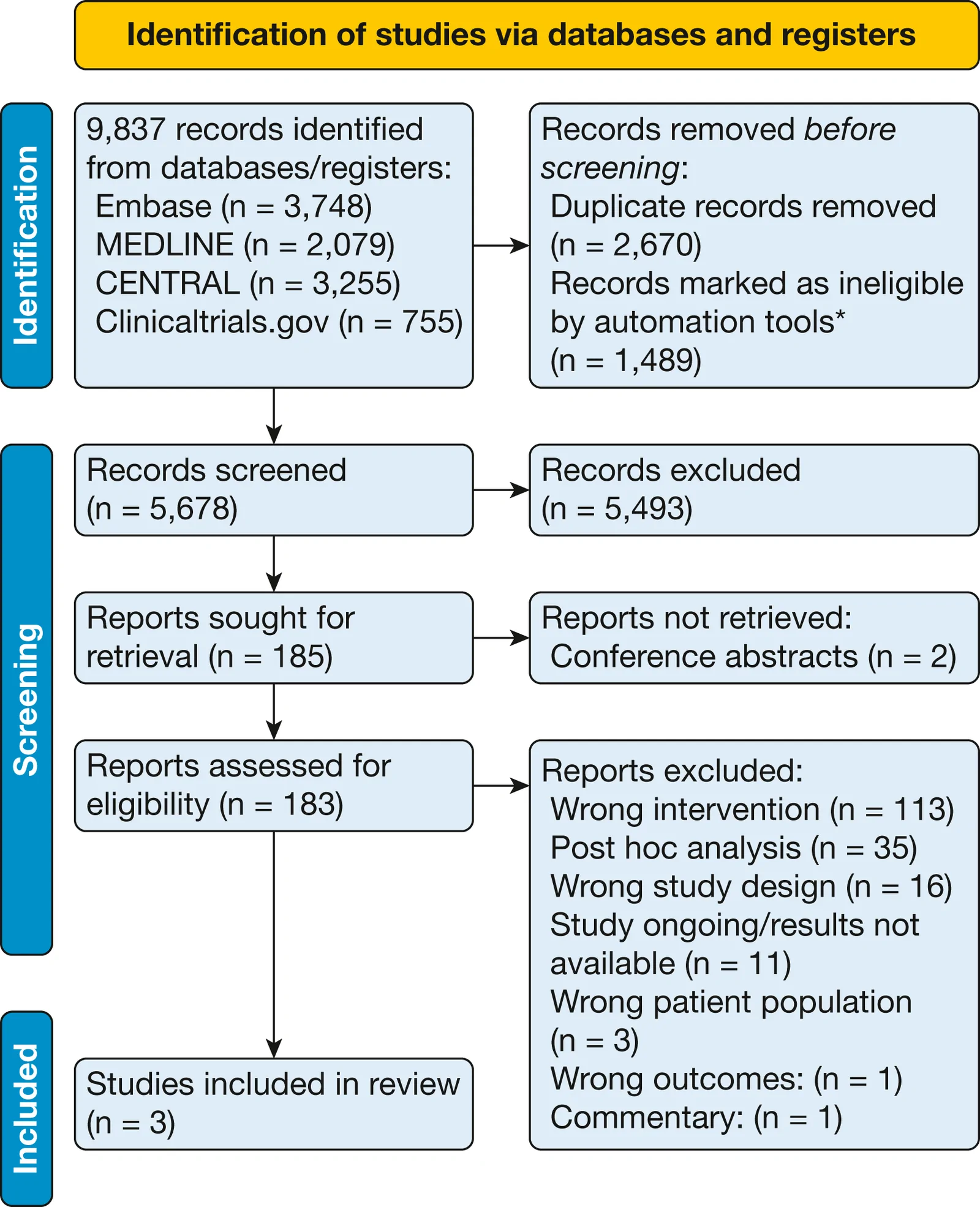
Literature search flow diagram. CENTRAL = Cochrane Central Register of Controlled Trials. ∗Covidence flags and can remove nonrandomized controlled trials using the Cochrane randomized controlled trial classifier.^14^

A total of 9,837 studies were identified by the searches. After removal of duplicates and references flagged as non-RCT by the Cochrane RCT classifier,^14^ 5,678 studies were screened for eligibility and 3 RCTs were included in this review as summarized in Figure 1. There was 1 RCT^15^ that included 2 eligible comparisons due to differing LAMA doses, which are henceforth reported separately. No study comparing HD or MD ICS/LABA/LAMA with LD ICS/LABA/LAMA was identified. Therefore, 4 treatment comparisons, and a combined total of 3,804 participants, comparing HD and MD ICS/LABA/LAMA were included. Participants in all studies were adults ≥ 18 years of age with uncontrolled asthma (ACQ > 1.5). The 3 RCTs all included similar proportions of participants who smoke/formerly smoked (20%-24%) and female participants (60%-63%). Two studies^7^^,^^16^ had as a recruitment criterion at least 1 severe exacerbation in the previous year. In the third study,^15^ 64% of participants reported ≥ 1 severe exacerbations in the previous year. The characteristics of the included studies are summarized in Table 1 and e-Table 1.

**Table 1:** Characteristics of Included Studies

Study	Sponsor	Baseline Treatment	Age Range, y	Duration, wk	No. of Analyzed Participants	Interventions
Lee et al^15^ CAPTAIN	GlaxoSmithKline	MD or HD ICS/LABA^b^	≥ 18	24-52^a^	1,623	FF/VI/UMEC 100/25/31.25 FF/VI/UMEC 200/25/31.25 FF/VI/UMEC 100/25/62.5 FF/VI/UMEC 200/25/62.5
Kerstjens et al^16^ IRIDIUM	Novartis	MD or HD ICS/LABA^b^	18-75	52	1,231	MF/IND/GLY 80/150/50 MF/IND/GLY 160/150/50
Gessner et al^7^ ARGON	Novartis	MD or HD ICS/LABA	≥ 18	24	950	MF/IND/GLY 80/150/50 MF/IND/GLY 160/150/50

The summary of the included study characteristics including equivalent Global Initiative for Asthma step and Global Initiative for Asthma ICS dose category. The number of analyzed participants is the number of participants randomized for the included study arms. See e-Table 1 for more detailed information on each study. ARGON = a randomised, phase IIIb, non-inferiority study; CAPTAIN = Clinical Study in Asthma Patients Receiving Triple Therapy in a Single Inhaler; FF = fluticasone furoate; GLY = Glycopyrronium; HD = high-dose; ICS = inhaled corticosteroid; IND = indacaterol; IRIDIUM = Once-daily, single-inhaler mometasone-indacaterol-glycopyrronium versus mometasone-indacaterol or twice-daily fluticasone-salmeterol in patients with inadequately controlled asthma; LABA = long-acting beta_2_-agonist; MD = medium-dose; MF = mometasone furoate; UMEC = umeclidinium; VI = vilanterol.

a All participants completed 24 wk, and this was the endpoint for the primary outcome analysis; however, some participants randomized during the initial recruitment period continued for a maximum of 52 wk. Exacerbation and safety data were extracted from the full 52-wk safety data and FEV_1_ and Asthma Control Questionnaire-7 data were extracted from the primary analysis data set at 24 wk.

b For CAPTAIN and IRIDIUM, although patients were enrolled on MD or HD ICS-LABA, they were all run-in on MD ICS-LABA (for 5 weeks and 2 weeks respectively).

Treatment periods were between 24 and 52 weeks in the 3 RCTs. In 1 RCT, the primary outcome analysis (change from trough FEV_1_) was at 24 weeks, but some participants remained on randomized treatment for up to 52 weeks.^15^ For this review, exacerbation and safety data were extracted from the full 52-week safety data, and data for the remaining outcomes were extracted from the primary analysis data set at 24 weeks. All 3 RCTs were sponsored by pharmaceutical companies. The individual study definitions of severe asthma exacerbation are described in e-Table 2.

### Risk of Bias and GRADE Assessment

The RCTs were all rated at a low risk of bias for severe asthma exacerbations and safety outcomes. For change from baseline FEV_1_ and ACQ-7, there were some concerns arising from the absence of reporting unadjusted data summaries for outcome data. None of these biases required downgrading of the certainty of their respective outcomes. A summary of the risk of bias assessments is available in e-Tables 3-5, and GRADE assessments are provided in e-Table 6. Statistical heterogeneity assessments did not indicate substantial heterogeneity for all pairwise comparisons, with point estimates of the I^2^ statistic between 0% and 53.2%, all with heterogeneity statistics which were not statistically significant at *P* < .05. This meant that estimates of effect based on fixed or random effects models were identical.

### Severe Exacerbations

**Figure 2:**
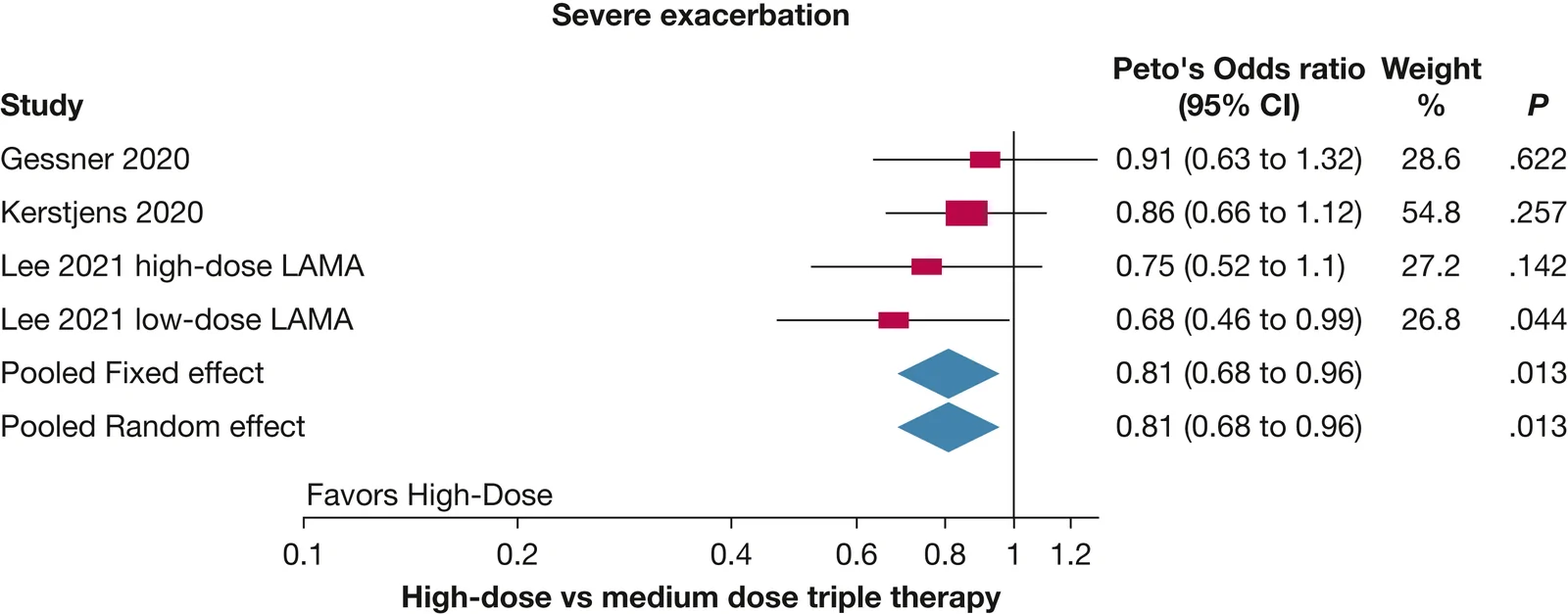
Forest plot of Peto OR for studies reporting the proportion of participants with ≥ 1 severe exacerbations, comparing medium-dose and high-dose inhaled corticosteroid/long-acting beta_2_-agonist/LAMA. Studies are ordered by their effect size, and the size of the squares (denoting the point estimate for Peto OR) is proportional to the study variance. LAMA = long-acting muscarinic antagonist.

All RCTs, totaling 3,804 participants, provided data on the number of participants with ≥ 1 severe exacerbations (Fig 2). The RCTs used comparable definitions to the joint American Thoracic Society and European Respiratory Society consensus statement (See e-appendix 2).^17^

Compared with MD ICS/LABA/LAMA, there was high-certainty evidence that HD ICS/LABA/LAMA reduced the odds of a severe exacerbation (Peto OR, 0.81; 95% CI, 0.68-0.96; *P* = .01).

In the planned secondary analysis, the associated absolute risk difference for the proportion of patients with ≥ 1 severe exacerbations was 3.04% (95% CI, 0.66-5.42), favoring the HD group. This is equivalent to a number needed to treat to prevent 1 severe exacerbation of 32.9.

### Change From Baseline FEV_1_

**Figure 3:**
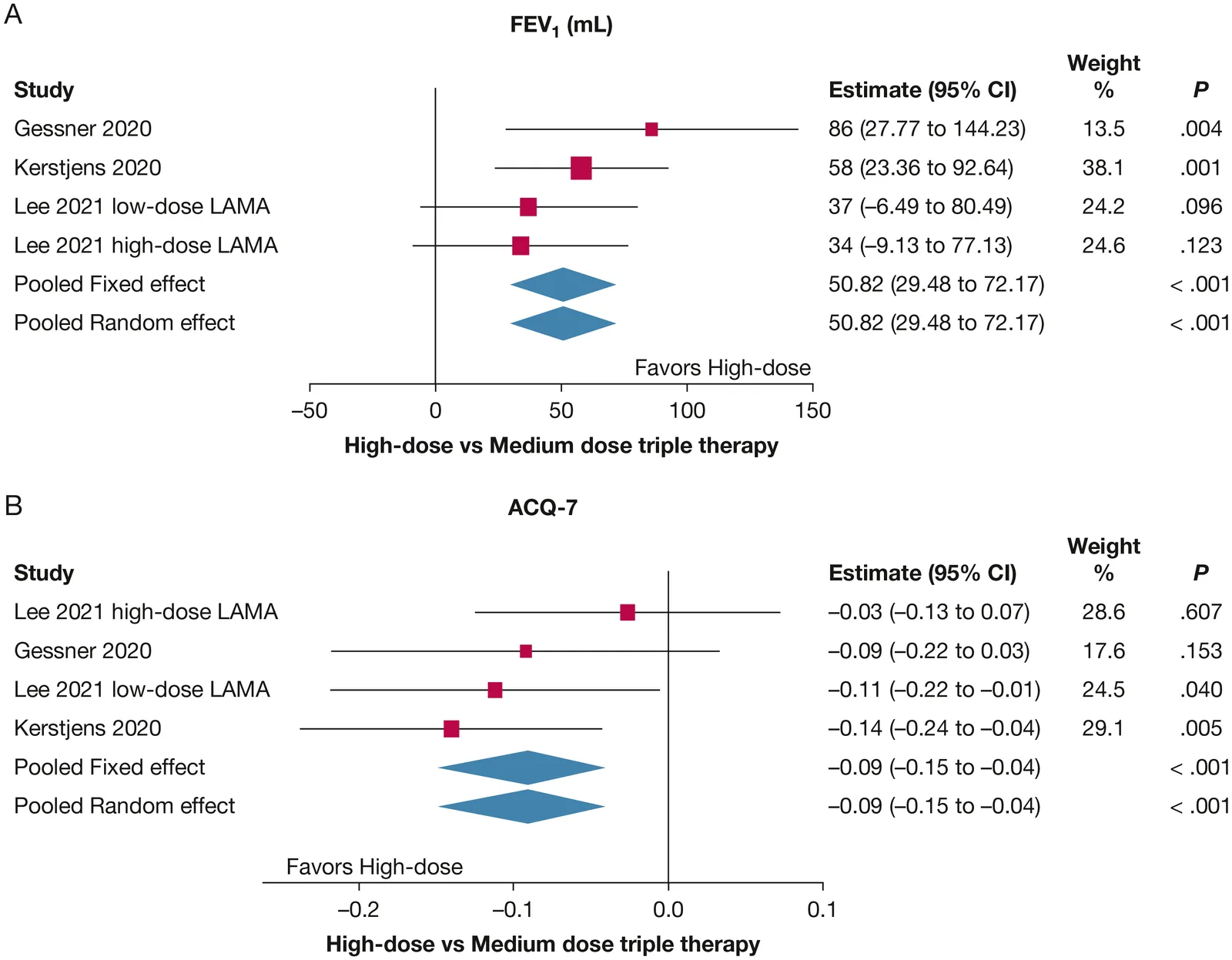
A-B, Forest plot of studies reporting (A) change from baseline FEV_1_ (mL), and (B) standardized mean difference for ACQ-7, comparing medium-dose and high-dose inhaled corticosteroid/long-acting beta_2_-agonist/LAMA. Studies are ordered by their effect size, and the size of the squares (denoting the study’s point estimate) is proportional to the study variance. ACQ-7 = Asthma Control Questionnaire-7; LAMA = long-acting muscarinic antagonist.

For the comparison of change in baseline FEV_1_ with HD and MD ICS/LABA/LAMA, the same 3 RCTS,^7^^,^^15^^,^^16^ reporting 4 treatment comparisons and including 3,448 participants, provided data (Fig 3A). No study reported raw data summaries for FEV_1_; therefore, least square means were used for all. For all studies, variance was estimated using the reported SE.

There was high-certainty evidence that HD ICS/LABA/LAMA resulted in a greater improvement in FEV_1_, with a mean difference of 50.8 mL (95% CI, 29.5-72.2 mL; *P* < .001). In the absence of important heterogeneity, the fixed and random effects models had the same effect size estimate. The upper 95% CI for this difference is well below the minimally clinically important difference (MCID) of between 170 and 250 mL depending on age and sex.^18^

### Change From Baseline ACQ-7

All 3 RCTs, totaling 4 treatment comparisons and 3,497 participants, reported change from baseline in the ACQ-7 (Fig 3B). For all studies, least square means were reported because unadjusted outcome data were not provided. Two RCTs had their variance estimated from the SE,^7^^,^^15^ and variance was estimated in the remaining study^16^ from point estimates of effect size and respective CIs, and the most conservative estimate was used.

There was high-certainty evidence that HD ICS/LABA/LAMA resulted in a greater reduction in ACQ-7 (improvement), with a mean difference of −0.09 (95% CI, −0.15 to −0.04; *P* < .001). The lower 95% CI for this difference is well below the MCID of 0.5.^19^

### ED Visits and Hospitalizations for Asthma

For the HD and MD ICS/LABA/LAMA comparison of participants with ≥ 1 ED visits for asthma, 2 treatment comparisons from 1 RCT^15^ contributed to the analysis with 1,623 participants. There were 10 and 20 ED visits in the HD and MD groups, respectively, resulting in a Peto OR of 0.51 (95% CI, 0.25-1.04; *P* = .07). For hospitalizations, 2,573 participants across 2 RCTs^7^^,^^15^ (with 3 treatment comparisons) contributed to the analysis. There were 13 and 20 hospital admissions for asthma in the HD and MD groups, respectively, resulting in a Peto OR of 0.65 (95% CI, 0.33-1.29; *P* = .22). The certainty of these results was downgraded due to imprecision (Table 2).

**Table 2:** Summary of Findings for Pairwise Comparisons of HD ICS/LABA/LAMA Compared With MD ICS/LABA/LAMA

Outcome	Participants (Studies)	Effect Estimate (95% CI)	Certainty	Clinical Interpretation
Severe exacerbations: ≥ 1	3,804 (3, with 4 treatment comparisons)	Peto OR, 0.81 (0.68-0.96)	⨁⨁⨁⨁ High	HD ICS/LABA/LAMA results in a modest reduction in the proportion of participants with ≥ 1 severe exacerbations
Change from baseline FEV_1_	3,448 (3, with 4 treatment comparisons)	Mean difference, 50.8 mL (29.5-72.2 mL)	⨁⨁⨁⨁ High	HD ICS/LABA/LAMA results in no clinically important difference in FEV_1_
Change from baseline ACQ-7	3,497 (3, with 4 treatment comparisons)	Mean difference, −0.09 (−0.15 to −0.04)	⨁⨁⨁⨁ High	HD ICS/LABA/LAMA results in no clinically important difference in ACQ-7
ED visit for asthma	1,623 (1, with 2 treatment comparisons)	Peto OR, 0.51 (0.25-1.04)	⨁⨁⨁ Moderate^a^	HD ICS/LABA/LAMA results in no statistically significant difference in ED visits for asthma
Hospitalization for asthma	2,573 (2, with 3 treatment comparisons)	Peto OR, 0.65 (0.33-1.29)	⨁⨁ Low^b^	HD ICS/LABA/LAMA results in no statistically significant difference in hospitalizations for asthma
SAE: ≥ 1	3,806 (3, with 4 treatment comparisons)	Peto OR, 1.04 (0.79-1.37)	⨁⨁⨁⨁ High	HD ICS/LABA/LAMA results in no difference in the proportion of participants with ≥ 1 SAEs
Deaths from asthma	3,806 (3, with 4 treatment comparisons)	No events	⨁⨁ Low^b^	Insufficient data to determine influence of HD ICS/LABA/LAMA on asthma mortality
All deaths	3,806 (3, with 4 treatment comparisons)	5 deaths reported in total	⨁⨁ Low^b^	Insufficient data to determine influence of HD ICS/LABA/LAMA on all-cause mortality

Certainty was assessed with Grading of Recommendations, Assessment, Development and Evaluations domains. ACQ-7 = Asthma Control Questionnaire-7; ED = emergency department; HD = high-dose; ICS = inhaled corticosteroid; LABA = long-acting beta_2_-agonist; LAMA = long-acting muscarinic antagonist; MD = medium-dose; SAE = serious adverse event.

a Marked down 2 levels for imprecision where there are few events and CI includes both appreciable benefit and appreciable harm.

b Marked down 1 level for imprecision when the optimal information size criterion is not met or if the 95% CI overlaps no effect.

### Serious Adverse Events and Deaths

All 3 RCTs^7^^,^^15^^,^^16^ totaling 3,806 participants were included in the comparison of participants with > 1 serious adverse event with HD and MD ICS/LABA/LAMA combination therapy. There was high-certainty evidence that there was no difference (Peto OR, 1.04; 95% CI, 0.79-1.37; *P* = .78). Similarly, data from all 3 RCTs with a total of 3,806 participants were included in analysis on deaths from asthma or any cause. There were no asthma-related deaths in any of the RCTs. Two RCTs reported 5 total deaths due to any cause. Given that a very large sample size would be required to identify the risk of death, the certainty of the findings was downgraded twice due to imprecision.

## Discussion

This study provides high-certainty evidence that maintenance HD ICS/LABA/LAMA therapy reduces the odds of severe exacerbation by around 20% compared with MD therapy. There were statistically significant benefits of HD therapy with lung function and asthma symptom control; however, the magnitude of the differences was below their respective MCIDs. For the risk of an ED visit or hospitalization due to asthma, the odds of 0.51 and 0.65 for HD vs MD therapy were not statistically significant; however, these estimates are limited by the small number of events, and do not rule out a clinically important effect. The absolute risk reduction in severe exacerbations with HD triple therapy was 3%, consistent with a number needed to treat of around 33. At both the individual and population level, this modest reduction in exacerbation risk must be balanced with the risks of systemic adverse effects associated with exposure to HD ICS, and may be only clinically relevant for patients with an estimated high baseline exacerbation risk, or in subgroups with high T2 status.

The 19% reduction in the odds of a severe exacerbation observed in this study, with a Peto OR of 0.81 (95% CI, 0.68-0.96), is almost identical to that seen in our parallel study comparing HD and MD ICS/LABA combination inhalers (Peto OR, 0.81; 95% CI, 0.67-0.98).^5^ This gives further confidence that we have identified an accurate measure of the true magnitude of effect of HD ICSs in patients with moderate to severe asthma. It supports our previous estimates from RCTs of the dose-response of ICSs in asthma, which reported that most efficacy with ICSs is achieved at a LD to MD.^2^^,^^3^ These findings are also consistent with real-world observational studies which reported minimal additional efficacy with stepping people up to HD ICSs from lower doses,^20^^,^^21^ suggesting one should not anticipate a large effect size when HD vs MD ICSs is used, irrespective of inhaler formulation.

Our findings are complemented by 2 systematic reviews and network meta-analyses^11^^,^^22^ which predominantly included indirect comparisons and used different inclusion criteria. One network meta-analysis^11^ pooled arms that had different devices, LABA doses, or LAMA doses, reducing the confidence that the observed risk ratio of 0.85 (95% CI, 0.72-1.01) reflected the true effect size due to the ICS doses. We also included lung function as a secondary outcome, which was not investigated in this network meta-analysis. The other network meta-analysis^22^ included the Triple in Asthma With Uncontrolled Patients on Medium Strength of ICS + LABA (TRIMARAN) and Triple in Asthma High Strength Versus ICS/LABA HS and Tiotropium (TRIGGER) RCTs,^23^ which were excluded from our analysis because participant allocation to MD or HD ICSs was based on prestudy ICS dose. Including these RCTs introduces selection bias because asthma severity may be systematically different between the MD and HD group. This may explain why the magnitude of severe exacerbation risk reduction was higher in this network meta-analysis, which reported a risk reduction of 35% (95% CI, 13%-51%) for HD vs MD ICS/LABA/LAMA inhaler therapy.^22^

Limitations of our analysis include the inclusion of only 3 trials, all of which had strict inclusion criteria that mandated FEV_1_ cutoffs and the presence of bronchodilator reversibility, potentially limiting the generalizability of these findings to the wider asthma population.^24^^,^^25^ Furthermore, the 3 studies only assessed inhaled fluticasone furoate and mometasone formulations, also potentially limiting generalizability to other ICSs.

In the assessment of risk, there were insufficient data to assess mortality and insufficient follow-up duration to comprehensively assess harm. The clinical trials included in the meta-analysis were not designed to assess the multiple risks of corticosteroid-related systemic side effects including reduced bone density, fractures, cataracts, type 2 diabetes, pneumonia, cardiovascular disease, and adrenal suppression.^1^^,^^4^^,^^26^, ^27^, ^28^, ^29^, ^30^ These effects are dose-dependent, and likely to be clinically important with long-term treatment within the range of HD ICS/LABA/LAMA therapy.^1^^,^^4^^,^^26^, ^27^, ^28^, ^29^, ^30^ For example, in the NORdic Dataset for aSThmA Research (NORDSTAR) cohort, hazard ratios linked to average daily HD ICS ranged from 1.16 for pneumonia to 1.70 for osteoporosis; sensitivity analysis excluding patients using oral corticosteroid (OCS) showed that HD ICS was still associated with an increased risk of systemic adverse effects.^29^ In the Comorbidity Burden in Severe and Nonsevere Asthma: A Nationwide Observational Study (FINASTHMA) cohort, the risks of pneumonia and osteoporosis with HD ICS was similar to those associated with 2 to 3 mg of prednisolone per day.^30^

The clinical conundrum is how to apply this modest benefit of HD ICSs, in dual and triple fixed dose combination inhalers, to clinical practice. One approach is outlined in the GINA 2025 report,^1^ that recommends consideration of a short-term (3-6 months) trial of HD ICSs in patients who continue to experience exacerbations despite MD ICS therapy. This approach is limited to patients having multiple exacerbations per year to have the capability to identify a response. Such a therapeutic trial of HD ICS therapy may be most likely to be beneficial for patients with high type 2 (T2) inflammatory status. A post hoc analysis from the Clinical Study in Asthma Patients Receiving Triple Therapy in a Single Inhaler (CAPTAIN) study, a 6-arm RCT which assessed fluticasone furoate-vilanterol across 2 doses, with or without umeclidinium, reported that HD fluticasone furoate resulted in a 6-fold greater reduction in severe exacerbations in patients with dual high T2 inflammation (high/high: blood eosinophil count ≥ 0.3 × 10^9^/L and fractional exhaled nitric oxide ≥ 50 ppb) compared with the dual T2-low/low subgroup (blood eosinophil count < 0.15 × 10^9^/L and fractional exhaled nitric oxide < 25 ppb).^15^ Unfortunately T2 inflammatory status was not reported for the other 2 RCTs so we could not stratify results by T2 status in this meta-analysis. Nonetheless, these findings suggest that escalation to HD ICSs should target patients with persistently high T2 biomarkers and be avoided in patients with low T2 biomarkers who face increased systemic risks without clear gain.

A complementary approach is to focus on clinician education and extension of systemic corticosteroid stewardship to the prescription of HD ICSs. In primary and secondary care patients across Canada, the US, Europe, Japan, and China whose management was captured in the Adelphi Real World Asthma Disease Specific Program, up to one-third were prescribed HD ICSs.^31^ Similarly, supratherapeutic ICS doses (> 1,600 μg budesonide equivalent daily, double that usually considered HD) were prescribed in 24% of patients initiating biologic therapy in Denmark, and physician-driven reductions in ICS dosing were rare even after 12 months of therapy.^32^ These studies demonstrate that prescribing practices are not in line with current GINA recommendations and risk exposing many patients to potential harms of HD ICS without appreciable benefit.

Importantly, there are numerous therapeutic alternatives to HD ICSs in patients with uncontrolled asthma, as recommended by GINA.^1^ MD ICS/formoterol maintenance and reliever therapy has at least similar efficacy to HD ICS/LABA while exposing patients to significantly less ICSs.^33^^,^^34^ Similarly, if an adult or adolescent receiving treatment at GINA step 3 or 4 has poorly controlled asthma, it is preferable to switch to the ICS/formoterol maintenance and reliever therapy regimen at the same step, compared with a step up or continuing the GINA treatment step with maintenance ICS/LABA together with short-acting beta-agonist (SABA) reliever therapy.^35^ Another therapeutic option is the use of MD ICS/LABA/LAMA, which has similar effects to HD ICS/LABA on exacerbations and FEV_1_ without additional ICS exposure.^22^ ICS/SABA reliever therapy in place of SABA reliever reduces the risk of severe asthma exacerbations among patients with uncontrolled moderate to severe asthma receiving a wide range of inhaled glucocorticoid-containing maintenance therapies.^36^ Long-term clinical data informing the corticosteroid related risks of ICS/SABA reliever therapy for patients already receiving HD ICSs is required to determine the risk/benefit profile of this regimen which potentially exposes patients to very high doses of ICSs. Finally, expanding the use of asthma biologics to patients exacerbating despite MD ICS-based treatments should also be a priority.

## Interpretation

Our results show that HD ICS/LABA/LAMA therapy is associated with about a 20% reduction in the odds of severe asthma exacerbation compared with MD ICS/LABA/LAMA therapy, a magnitude of effect that is almost identical to that observed with HD vs MD ICS/LABA. At the individual and population level, this effect is likely to be clinically relevant to patients with an elevated baseline exacerbation risk, such as those with frequent severe exacerbations, and in those with high T2 status, supporting the concept of targeted HD ICS-based therapy. In clinical practice, consideration needs to be given to the numerous alternative regimens to HD ICS/LABA/LAMA therapy that have similar or greater efficacy and reduce exposing patients to the risks associated with HD ICS therapy.

## Funding/Support

The Medical Research Institute of New Zealand receives independent research organization funding from the 10.13039/501100001505Health Research Council of New Zealand to perform research activities including this systematic review and meta-analysis.

## Financial/Nonfinancial Disclosures

The authors have reported to *CHEST Pulmonary* the following: R. W. B. has received institutional research funding from AstraZeneca and Teva; personal fees from AstraZeneca, Avillion, Teva Pharmaceuticals, and Cipla, outside the submitted work; is chair of the Asthma Foundation of New Zealand adolescent and adult asthma guidelines group; and was a member of the board of directors of the Global Initiative for Chronic Obstructive Lung Disease. None declared (S. W., R. C., R. S., F. L., J. K., M. W., J. H. N.).

## Supplementary Data

e-Online Data
