# Supplementary material for: Medium-Dose Compared With High-Dose Inhaled Corticosteroids in Combination Triple Inhaler Therapy for Asthma: A Systematic Review and Meta-Analysis
Source: CHEST Pulm. 2026 Feb 21;4(3):100240. doi: 10.1016/j.chpulm.2026.100240 (PMC13534840; doi:10.1016/j.chpulm.2026.100240)
Supplement: Supplementary file 1 [file mmc1.docx]

**MEDIUM COMPARED TO HIGH DOSE INHALED CORTICOSTEROIDS IN COMBINATION TRIPLE INHALER THERAPY FOR ASTHMA: A SYSTEMATIC REVIEW AND META-ANALYSIS**

Samantha Warhurst; Ryan Cullen; Ross Sayers; Francesca Lynch; Joseph Kulathinal; Mark Weatherall; Richard W Beasley; Jonathan H Noble

**ONLINE DATA SUPPLEMENT**

Contents

[E-Methods 1. Search strategies	3](#_Toc198623995)

[Table E1: Additional characteristics for included studies.	6](#_Toc198623996)

[Table E2: Included study definitions of severe asthma exacerbations.	7](#_Toc198623997)

[Table E3: Risk of bias assessment for severe exacerbations and safety outcomes.	8](#_Toc198623998)

[Table E4: Risk of bias assessment for change from baseline FEV1.	9](#_Toc198623999)

[Table E5: Risk of bias assessment for change from baseline validated asthma symptom questionnaire.	11](#_Toc198624000)

[Table E6: Grading of Recommendations, Assessment, Development, and Evaluation (GRADE) assessment for each outcome.	12](#_Toc198624001)

[E-Results 1: Extracted data summaries and statistical analysis	13](#_Toc198624002)

[Number of participants with 1 or more severe exacerbations	13](#_Toc198624003)

[Number of participants with 1 or more SAE	15](#_Toc198624004)

[Hospital admission for asthma	16](#_Toc198624005)

[ED visits for asthma	17](#_Toc198624006)

[Deaths from asthma	19](#_Toc198624007)

[All deaths	20](#_Toc198624008)

[Change from baseline FEV1	21](#_Toc198624009)

[Change from baseline in patient reported outcome measure (ACQ or ACT)	23](#_Toc198624010)

E-Methods 1. Search strategies**.** All searches were repeated a final time to include studies up to 31 December 2024 prior to data extraction.

| Ovid MEDLINE(R) <1946 to June Week 4 2024> | | |
| --- | --- | --- |
| 1 | (asthma* or wheeze*).mp. | 195826 |
| 2 | (fluticasone* or budesonide or beclometasone* or mometasone or ciclesonide or triamcinolone* or flunisolide).mp. | 23876 |
| 3 | (formoterol or eformoterol or salmeterol or vilanterol or indacaterol).mp. | 5630 |
| 4 | 2 and 3 | 2647 |
| 5 | ((ICS adj2 LABA) or (inhal* adj corticosteroid* adj2 long-acting beta* agonist) or (inhal* adj corticosteroid* adj2 long acting beta* agonist) or (ICS adj2 long-acting beta*agonist) or (ICS adj2 long acting beta*agonist)).mp. | 1221 |
| 6 | 4 or 5 | 3437 |
| 7 | (aclidinium or glycopyrronium or tiotropium or umeclidinium).mp. | 2467 |
| 8 | 2 and 3 and 7 | 436 |
| 9 | (ICS#LAMA#LABA or ICS#LABA#LAMA or (ICS adj2 LAMA adj2 LABA) or (ICS adj2 LABA adj2 LAMA) or (inhal* adj corticosteroid* adj2 long-acting beta* agonist adj2 long-acting muscarinic antagonist) or (inhal* adj corticosteroid* adj2 long acting beta* agonist adj2 long acting muscarinic antagonist) or (ICS adj2 long-acting beta*agonist adj2 long-acting muscarinic antagonist) or (ICS adj2 long acting beta*agonist adj2 long acting muscarinic antagonist)).mp. | 257 |
| 10 | 8 or 9 | 640 |
| 11 | 1 and 6 | 2231 |
| 12 | 1 and 10 | 163 |
| 13 | 11 or 12 | 2234 |
| 14 | exp randomized controlled trial/ | 616995 |
| 15 | controlled clinical trial.pt. | 95538 |
| 16 | randomized.ab. | 560649 |
| 17 | placebo.ab. | 226236 |
| 18 | drug therapy.fs. | 2707837 |
| 19 | randomly.ab. | 364296 |
| 20 | trial.ab. | 606261 |
| 21 | groups.ab. | 2266399 |
| 22 | 14 or 15 or 16 or 17 or 18 or 19 or 20 or 21 | 5426291 |
| 23 | exp animals/ not humans.sh. | 5234286 |
| 24 | 22 not 23 | 4676916 |
| 25 | 13 and 24 | 2014 |

| Embase <1974 to 2024 July 08> | | |
| --- | --- | --- |
| 1 | exp Asthma/ | 314469 |
| 2 | (asthma* or wheeze*).mp. | 375522 |
| 3 | 1 or 2 | 375903 |
| 4 | (fluticasone* or budesonide or beclometasone* or mometasone or ciclesonide or triamcinolone* or flunisolide).mp. | 90633 |
| 5 | (formoterol or eformoterol or salmeterol or vilanterol or indacaterol).mp. | 21796 |
| 6 | 4 and 5 | 14496 |
| 7 | ((ICS adj2 LABA) or (inhal* adj corticosteroid* adj2 long-acting beta* agonist) or (inhal* adj corticosteroid* adj2 long acting beta* agonist) or (ICS adj2 long-acting beta*agonist) or (ICS adj2 long acting beta*agonist)).mp. | 3728 |
| 8 | 6 or 7 | 16827 |
| 9 | (aclidinium or glycopyrronium or tiotropium or umeclidinium).mp. | 17678 |
| 10 | 4 and 5 and 9 | 3685 |
| 11 | (ICS#LAMA#LABA or ICS#LABA#LAMA or (ICS adj2 LAMA adj2 LABA) or (ICS adj2 LABA adj2 LAMA) or (inhal* adj corticosteroid* adj2 long-acting beta* agonist adj2 long-acting muscarinic antagonist) or (inhal* adj corticosteroid* adj2 long acting beta* agonist adj2 long acting muscarinic antagonist) or (ICS adj2 long-acting beta*agonist adj2 long-acting muscarinic antagonist) or (ICS adj2 long acting beta*agonist adj2 long acting muscarinic antagonist)).mp. | 863 |
| 12 | 10 or 11 | 4286 |
| 13 | 3 and 8 | 10865 |
| 14 | 3 and 12 | 1632 |
| 15 | 13 or 14 | 10875 |
| 16 | Clinical trial/ | 1085020 |
| 17 | Randomized controlled trial/ | 830232 |
| 18 | Randomization/ | 99540 |
| 19 | Single blind procedure/ | 55384 |
| 20 | Double blind procedure/ | 220845 |
| 21 | Crossover procedure/ | 78659 |
| 22 | Placebo/ | 414831 |
| 23 | Randomized controlled trial$.tw. | 350629 |
| 24 | Rct.tw. | 58407 |
| 25 | Random allocation.tw. | 2724 |
| 26 | Randomly allocated.tw. | 48116 |
| 27 | Allocated randomly.tw. | 3069 |
| 28 | (allocated adj2 random).tw. | 967 |
| 29 | Single blind$.tw. | 33444 |
| 30 | Double blind$.tw. | 253438 |
| 31 | ((treble or triple) adj blind$).tw. | 2153 |
| 32 | Placebo$.tw. | 381704 |
| 33 | Prospective study/ | 925984 |
| 34 | 16 or 17 or 18 or 19 or 20 or 21 or 22 or 23 or 24 or 25 or 26 or 27 or 28 or 29 or 30 or 31 or 32 or 33 | 2890490 |
| 35 | Case study/ | 101744 |
| 36 | Case report.tw. | 575808 |
| 37 | Abstract report/ or letter/ | 1335947 |
| 38 | 35 or 36 or 37 | 1997152 |
| 39 | 34 not 38 | 2822393 |
| 40 | 15 and 39 | 3667 |

| Cochrane Register of Controlled Trials (CENTRAL) - 27/08/2024 | | |
| --- | --- | --- |
| ID | Search | Hits |
| #1 | (asthma):ti,ab,kw OR (wheeze):ti,ab,kw | 36870 |
| #2 | MeSH descriptor: [Asthma] explode all trees | 15066 |
| #3 | #1 OR #2 with Cochrane Library publication date Between Jan 1946 and Jul 2024, in Cochrane Reviews, Cochrane Protocols, Trials, Clinical Answers, Editorials, Special Collections | 36878 |
| #4 | fluticasone OR budesonide OR beclometasone OR mometasone OR ciclesonide OR trimamcinolone OR flunisolide | 13235 |
| #5 | formoterol OR eformoterol OR salmeterol OR vilanterol OR indacaterol | 7849 |
| #6 | #4 AND #5 | 4651 |
| #7 | ("inhaled corticosteroid" AND LABA):ti,ab,kw OR (ICS AND LABA):ti,ab,kw OR (ICS AND long acting beta agonist):ti,ab,kw | 1639 |
| #8 | #6 OR #7 | 5528 |
| #9 | aclidinium OR glycopyrronium OR tiotropium OR umeclidinium | 4030 |
| #10 | MeSH descriptor: [Muscarinic Antagonists] explode all trees | 1258 |
| #11 | #9 OR #10 | 4986 |
| #12 | #4 AND #5 AND #11 | 946 |
| #13 | (ICS AND LAMA AND LABA):ti,ab,kw OR ("inhaled corticosteroid" AND LABA AND LABA):ti,ab,kw OR ("inhaled corticosteroid" AND long acting beta agonist AND long acting muscarinic antagonist):ti,ab,kw | 844 |
| #14 | #12 OR #13 | 1559 |
| #15 | #3 AND #8 | 3302 |
| #16 | #3 AND #14 | 567 |
| #17 | #15 OR #16 | 3303 |
| #18 | #17 with 'Airways' in Cochrane Groups | 1711 |
| #19 | #17 OR #18 | 3303 |

| ClinicalTrials.Gov - 14/10/24 | |
| --- | --- |
| asthma OR wheeze \| Other terms: inhaled corticosteroids \(ICS\) and long-acting β2-agonist \(LABA\) OR inhaled corticosteroids \(ICS\) OR fluticasone OR budesonide OR beclometasone OR mometasone OR ciclesonide OR triamcinolone OR flunisolide OR formoterol OR eformoterol OR salmeterol OR vilanterol OR indacaterol OR aclidinium OR glycopyrronium OR tiotropium OR umeclidinium \| Phase: 2, 3, 4 \| Interventional studies \| Study start from 01/01/1946 to 07/31/2024 | 746 |

# Table E1: Additional characteristics for included studies

Abbreviations: BD, twice daily; HD, high dose; ICS, inhaled corticosteroids; IND, indacaterol; LABA, long-acting beta2-agonist; MD, medium dose; MF, mometasone furoate; NR, not reported; OD, once daily; SAL, salmeterol; VI, vilanterol.

| **Study** | **Participants, N** | **Countries** | **Female N (%)** | **Current or ex-smokers N (%)** | **N (%) treated with LD ICS at baseline** | **N (%) treated with MD ICS at baseline** | **N (%) treated with HD ICS at baseline** | **Study aim** |
| --- | --- | --- | --- | --- | --- | --- | --- | --- |
| Lee 2021 | 1623 | Argentina, Australia, Canada, Germany, Italy, Japan, Korea, Netherlands, Poland, Romania, Russia, South Africa, Spain, United Kingdom, United States | 1008 (62) | 332 (20) | 0 (0) | 1090 (67) | 533 (33) | To investigate the efficacy and safety of the single-inhaler triple combination of fluticasone furoate/umeclidinium/vilanterol (FF/UMEC/VI) in patients with uncontrolled asthma on medium-high dose ICS/LABA. To explore the potential for a treatable traits approach to inhaled therapy, they also assessed whether the effects of increasing FF dose or adding UMEC on lung function and exacerbations were related to baseline type 2 airway inflammation biomarkers |
| Kerstjens 2020 | 1239 | Argentina, Austria, Belgium, Bulgaria, Canada, Chile, China, Colombia, Croatia, Denmark, Estonia, Finland, France, Germany, Greece, Hungary, India, Ireland, Israel, Italy, Japan, Jordon, Latvia, Lebanon, Lithuania, Mexico, Netherlands, Peru, Phillipines, Poland, Portugal, Romania, Russia, Slovakia, South Africa, Spain, Sweden, Switzerland, Thailand, United Kingdom, Vietnam | 743 (60) | 245 (20) | 6 (0) | 765 (61) | 463 (37) | The goal of the phase 3 IRIDIUM study was to assess the efficacy and safety of medium-dose and high-dose MF/IND/GLY once daily versus medium-dose and high-dose MF/IND once daily, to show the beneficial effect of the addition of glycopyrronium, and to compare the benefits of MF/IND/GLY with a well-established ICS/LABA combination, high-dose fluticasone-salmeterol (FLU/SAL) twice daily, in adult patients with inadequately controlled asthma. |
| Gessner 2020 | 950 | Argentina, Chile, Colombia, Czech Republic, Germany, Greece, Hungary, India, Israel, Mexico, Peru, Poland, Russia, Serbia, Slovakia, South Africa, Spain, Taiwan, Turkey, Vietnam | 595 (63) | 226 (24) | 0 (0) | 459 (48) | 481 (51) | To compare the ef­ficacy and safety of high and medium dose IND/GLY/MF high (150/50/160 μg and150/50/80 μg once daily) compared with concurrent administration of standard of care SAL/FLU high-dose (50/500 μg) twice daily (b.i.d.) + TIO (5 μg) o.d. in patients with uncontrolled asthma who were already on medium or high dose ICS/LABA inhaled therapy. |

# Table E2: Included study definitions of severe asthma exacerbations

| **Study** | **Study definition of severe asthma exacerbation used for meta-analysis comparisons** |
| --- | --- |
| Lee 2021 | Exacerbation of asthma requiring admission to hospital or a visit to an emergency department due to the need for systemic corticosteroids, or asthma deterioration requiring systemic corticosteroid use (or doubling of the current maintenance systemic corticosteroid dose) for a≥ 3 days. |
| Kerstjens 2020 | An aggravation of asthma symptoms that requires systemic corticosteroids for ≥3 consecutive days or a need for an emergency room visit, hospitalisation owing to asthma, or death due to asthma. |
| Gessner 2020 | An aggravation of asthma symptoms (like shortness of breath, cough, wheezing, or chest tightness) that required systemic corticosteroids for at least three consecutive days and/or a need for an emergency room visit (or local equivalent structure), or hospitalisation due to asthma or death due to asthma. |

Table E3: Risk of bias assessment for severe exacerbations and safety outcomes.

Assessed using Cochrane risk of bias tool.

Abbreviations: ED, emergency department; hosp, hospital admissions; SAEs, serious adverse events

| **Study** | **Outcomes** | **D1** | **D2** | **D3** | **D4** | **D5** | **Overall** |  |  |
| --- | --- | --- | --- | --- | --- | --- | --- | --- | --- |
| Lee 2021 | Severe exacerbations, SAEs, hospitalisations, ED, asthma deaths, all deaths |  |  |  |  |  |  |  | Low risk |
| Kerstjens 2020 | Severe exacerbations, SAEs, asthma deaths, all deaths |  |  |  |  |  |  |  | Some concerns |
| Gessner, 2020 | Severe exacerbations, SAEs, hospitalisations, asthma deaths, all deaths |  |  |  |  |  |  |  | High risk |

Table E4: Risk of bias assessment for change from baseline FEV_1_. Assessed using Cochrane risk of bias tool

Abbreviations: FEV_1_, forced expiratory volume in 1 second

| **Study** | **Outcome** | **D1** | **D2** | **D3** | **D4** | **D5** | **Overall** |  |  |  |
| --- | --- | --- | --- | --- | --- | --- | --- | --- | --- | --- |
| Lee, 2021 | Change from baseline trough FEV_1_ |  |  |  |  |  |  |  | Low risk |  |
| Kerstjens, 2020 | Change from baseline trough FEV_1_ |  |  |  |  |  |  |  | Some concerns |  |
| Gessner, 2020 | Change from baseline in trough FEV_1_ |  |  |  |  |  |  |  | High risk |  |

Table E5: Risk of bias assessment for change from baseline validated asthma symptom questionnaire

Assessed using Cochrane risk of bias tool

Abbreviations: ACQ, Asthma Control Questionnaire; ACT, Asthma Control Test

| **Study** | **Outcome** | **D1** | **D2** | **D3** | **D4** | **D5** | **Overall** |  |  |
| --- | --- | --- | --- | --- | --- | --- | --- | --- | --- |
| Lee, 2021 | Change from baseline ACQ-7 |  |  |  |  |  |  |  | Low risk |
| Kerstjens, 2020 | Change from baseline ACQ-7 |  |  |  |  |  |  |  | Some concerns |
| Gessner, 2020 | Change from baseline ACQ-7 |  |  |  |  |  |  |  | High risk |
|  |  |  |  |  |  |  |  |  |  |

Table E6: Grading of Recommendations, Assessment, Development, and Evaluation (GRADE) assessment for each outcome

*Marked down two levels for imprecision where there are few events and confidence interval include both appreciable benefit and appreciable harm. #Marked down one level for imprecision when the optimal information size criterion is not met or if the 95% confidence interval overlaps no effect.

Abbreviations; ED, emergency department; FEV_1_, forced expiratory volume in 1 second; HD, high-dose; ICS/LABA, inhaled corticosteroid/long-acting beta2-agonist/long-acting muscarinic antagonist; MD, medium-dose; SAE, serious adverse event

| Outcome | Comparison | Risk of bias | Inconsistency | Indirectness | Imprecision | Publication bias | Final rating | Final rating, symbol |
| --- | --- | --- | --- | --- | --- | --- | --- | --- |
| Severe exacerbations: one or more | HD v MD ICS/LABA/LAMA | not serious | not serious | not serious | not serious | undetected | High | ⨁⨁⨁⨁ |
| Change from baseline FEV_1_ | HD v MD ICS/LABA/LAMA | not serious | not serious | not serious | not serious | undetected | High | ⨁⨁⨁⨁ |
| Change from baseline ACQ-7 | HD v MD ICS/LABA/LAMA | not serious | not serious | not serious | not serious | undetected | High | ⨁⨁⨁⨁ |
| ED admission due to asthma | HD v MD ICS/LABA/LAMA | not serious | not serious | not serious | serious# | undetected | Moderate | ⨁⨁⨁ |
| Hospitalisation due to asthma | HD v MD ICS/LABA/LAMA | not serious | not serious | not serious | very serious* | undetected | Low | ⨁⨁ |
| SAE: one or more | HD v MD ICS/LABA/LAMA | not serious | not serious | not serious | not serious# | undetected | High | ⨁⨁⨁⨁ |
| Death from asthma | HD v MD ICS/LABA/LAMA | not serious | not serious | not serious | very serious* | undetected | Low | ⨁⨁ |
| All cause death | HD v MD ICS/LABA/LAMA | not serious | not serious | not serious | very serious* | undetected | Low | ⨁⨁ |

# E-Results 1: Extracted data summaries and statistical analysis

## Number of participants with 1 or more severe exacerbations

Extracted data summaries

|  | **N/N (%)** | |
| --- | --- | --- |
| **Study** | **High dose** | **Medium dose** |
| Gessner 2020 | 64/476 (13.4) | 69/474 (14.6) |
| Kerstjens 2020 | 134/615 (21.8) | 151/616 (24.5) |
| Lee 2021 high-dose LAMA | 57/408 (14) | 72/406 (17.7) |
| Lee 2021 low-dose LAMA | 53/404 (13.1) | 74/405 (18.3) |

Study and Pooled estimates by Peto’s method

| **Study** | **Odds ratio (95% CI)** | **P** |
| --- | --- | --- |
| Gessner 2020 | 0.91 (0.63 to 1.32) | 0.62 |
| Kerstjens 2020 | 0.86 (0.66 to 1.12) | 0.26 |
| Lee 2021 high-dose LAMA | 0.75 (0.52 to 1.1) | 0.14 |
| Lee 2021 low-dose LAMA | 0.68 (0.46 to 0.99) | 0.04 |
| Pooled Fixed effect | 0.81 (0.68 to 0.96) | 0.01 |
| Pooled Random effect | 0.81 (0.68 to 0.96) | 0.01 |

Note a higher odds ratio means more likely to have an event.

Heterogeneity and I-squared statistic

| **Chi-square (DF)** | **P** | **I-squared (95% CI)** |
| --- | --- | --- |
| 1.6 (3) | 0.67 | 0 (0 to 70.8) |

Publication bias

| **Method** | **Test statistic** |
| --- | --- |
| Correlation coefficient | -0.67  P=0.17 |

## Number of participants with 1 or more SAE

Extracted data summaries

|  | **N/N (%)** | |
| --- | --- | --- |
| **Study** | **High dose** | **Medium dose** |
| Gessner 2020 | 18/476 (3.8) | 14/474 (3.0) |
| Kerstjens 2020 | 46/616 (7.5) | 49/617 (7.9) |
| Lee 2021 high-dose LAMA | 21/408 (5.1) | 23/406 (5.7) |
| Lee 2021 low-dose LAMA | 23/404 (5.7) | 18/405 (4.4) |

Study and Pooled estimates by Peto’s method

| **Study** | **Odds ratio (95% CI)** | **P** |
| --- | --- | --- |
| Gessner 2020 | 1.29 (0.64 to 2.61) | 0.48 |
| Kerstjens 2020 | 0.94 (0.62 to 1.42) | 0.76 |
| Lee 2021 high-dose LAMA | 0.90 (0.49 to 1.66) | 0.74 |
| Lee 2021 low-dose LAMA | 1.30 (0.69 to 2.43) | 0.42 |
| Pooled Fixed effect | 1.04 (0.79 to 1.37) | 0.78 |
| Pooled Random effect | 1.04 (0.79 to 1.37) | 0.78 |

Note a higher odds ratio means more likely to have an event.

Heterogeneity and I-squared statistic

| **Chi-square (DF)** | **P** | **I-squared (95% CI)** |
| --- | --- | --- |
| 1.3 (3) | 0.73 | 0 (0 to 64.1) |

### Publication bias

| **Method** | **Test statistic** |
| --- | --- |
| Correlation coefficient | 0.67  P=0.17 |

Forest plot

## Hospital admission for asthma

Extracted data summaries

|  | **N/N (%)** | |
| --- | --- | --- |
| **Study** | **High dose** | **Medium dose** |
| Gessner 2020 | 3/476 (0.6) | 4/474 (0.8) |
| Lee 2021 high-dose LAMA | 6/408 (1.5) | 8/406 (2.0) |
| Lee 2021 low-dose LAMA | 4/404 (1.0) | 8/405 (2.0) |

Study and Pooled estimates by Peto’s method

| **Study** | **Odds ratio (95% CI)** | **P** |
| --- | --- | --- |
| Gessner 2020 | 0.75 (0.17 to 3.30) | 0.70 |
| Lee 2021 high-dose LAMA | 0.74 (0.26 to 2.14) | 0.58 |
| Lee 2021 low-dose LAMA | 0.51 (0.16 to 1.59) | 0.25 |
| Pooled Fixed effect | 0.65 (0.33 to 1.29) | 0.22 |
| Pooled Random effect | 0.65 (0.33 to 1.29) | 0.22 |

Note a higher odds ratio means more likely to have an event.

Heterogeneity and I-squared statistic

| **Chi-square (DF)** | **P** | **I-squared (95% CI)** |
| --- | --- | --- |
| 0.27 (2) | 0.87 | 0 (0 to 23.2) |

Publication bias

| **Method** | **Test statistic** |
| --- | --- |
| Correlation coefficient | -0.33  P=0.60 |

Forest plot

## ED visits for asthma

Extracted data summaries

|  | **N/N (%)** | |
| --- | --- | --- |
| **Study** | **High dose** | **Medium dose** |
| Lee 2021 high-dose LAMA | 4/408 (1) | 8/406 (2) |
| Lee 2021 low-dose LAMA | 6/404 (1.5) | 12/405 (3) |

Study and Pooled estimates by Peto’s method

| **Study** | **Odds ratio (95% CI)** | **P** |
| --- | --- | --- |
| Lee 2021 high-dose LAMA | 0.51 (0.16 to 1.58) | 0.24 |
| Lee 2021 low-dose LAMA | 0.51 (0.20 to 1.29) | 0.15 |
| Pooled Fixed effect | 0.51 (0.25 to 1.04) | 0.07 |
| Pooled Random effect | 0.51 (0.25 to 1.04) | 0.07 |

Note a higher odds ratio means more likely to have an event.

Heterogeneity and I-squared statistic

| **Chi-square (DF)** | **P** | **I-squared (95% CI)** |
| --- | --- | --- |
| 0 (1) | >0.99 | 0 (0 to NA) |

Publication bias

| **Method** | **Test statistic** |
| --- | --- |
| Correlation coefficient | NA |

Forest plot

## Deaths from asthma

Extracted data summaries

|  | **N/N (%)** | |
| --- | --- | --- |
| **Study** | **High dose** | **Medium dose** |
| Gessner 2020 | 0/476 (0) | 0/474 (0) |
| Kerstjens 2020 | 0/616 (0) | 0/617 (0) |
| Lee 2021 high-dose LAMA | 0/408 (0) | 0/406 (0) |
| Lee 2021 low-dose LAMA | 0/408 (0) | 0/406 (0) |

Study and Pooled estimates by Peto’s method

Note no studies with non-zero cell counts.

Heterogeneity and I-squared statistic

| **Chi-square (DF)** | **P** | **I-squared (95% CI)** |
| --- | --- | --- |
| NA | NA | NA |

Publication bias

| **Method** | **Test statistic** |
| --- | --- |
| Correlation coefficient | NA |

Forest plot

Note no studies with non-zero cell counts

## All deaths

Extracted data summaries

|  | **N/N (%)** | |
| --- | --- | --- |
| **Study** | **High dose** | **Medium dose** |
| Gessner 2020 | 0/476 (0) | 0/474 (0) |
| Kerstjens 2020 | 2/616 (0.3) | 1/617 (0.2) |
| Lee 2021 high-dose LAMA | 0/408 (0) | 0/406 (0) |
| Lee 2021 low-dose LAMA | 0/404 (0) | 2/405 (0.5) |

Study and Pooled estimates by Peto’s method

| **Study** | **Odds ratio (95% CI)** | **P** |
| --- | --- | --- |
| Gessner 2020 | NA | NA |
| Kerstjens 2020 | 1.95 (0.2 to 18.81) | 0.56 |
| Lee 2021 high-dose LAMA | NA | NA |
| Lee 2021 low-dose LAMA | 0.14 (0.01 to 2.17) | 0.16 |
| Pooled Fixed effect | 0.67 (0.12 to 3.88) | 0.66 |
| Pooled Random effect | 0.61 (0.07 to 5.59) | 0.66 |

Heterogeneity and I-squared statistic

| **Chi-square (DF)** | **P** | **I-squared (95% CI)** |
| --- | --- | --- |
| 2.1 (1) | 0.14 | 53.2 (0 to 88.4) |

Publication bias

| **Method** | **Test statistic** |
| --- | --- |
| Correlation coefficient | NA |

Forest plot

Note very sparse data with extremely wide confidence intervals, plot not shown

## Change from baseline FEV_1_

Extracted data summaries

|  | **High dose** | | **Medium dose** | |
| --- | --- | --- | --- | --- |
| **Study** | **N** | **Mean (SD)** | **N** | **Mean (SD)** |
| Gessner 2020 | 438 | 334 (439.5) | 420 | 248 (430.4) |
| Kerstjens 2020 | 532 | 321 (285.4) | 512 | 263 (285.4) |
| Lee 2021 high-dose LAMA | 391 | 168 (307.7) | 390 | 134 (307.3) |
| Lee 2021 low-dose LAMA | 384 | 157 (304.9) | 381 | 120 (308.7) |

Study and pooled estimates by inverse variance weighted method

| **Study** | **Difference (95% CI)** | **P** |
| --- | --- | --- |
| Gessner 2020 | 86 (27.8 to 144.2) | 0.004 |
| Kerstjens 2020 | 58 (23.4 to 92.6) | 0.001 |
| Lee 2021 high-dose LAMA | 34 (-9.1 to 77.1) | 0.12 |
| Lee 2021 low-dose LAMA | 37 (-6.5to 80.5) | 0.10 |
| Pooled Fixed effect | 50.8 (29.5 to 72.2) | <0.001 |
| Pooled Random effect | 50.8 (29.5 to 72.2) | <0.001 |

Heterogeneity and I-squared statistic

| **Chi-square (DF)** | **P** | **I-squared (95% CI)** |
| --- | --- | --- |
| 2.5 (3) | 0.47 | 0 (0 to 81.9) |

Publication bias

| **Method** | **Test statistic** |
| --- | --- |
| Correlation coefficient | 0.33  P=0.50 |

## Change from baseline in Asthma Control Questionnaire-7

Extracted data summaries

|  | **High dose** | | **Medium dose** | |
| --- | --- | --- | --- | --- |
| **Study** | **N** | **Mean (SD)** | **N** | **Mean (SD)** |
| Gessner 2020 | 454 | -1.2 (1.0) | 447 | -1.1 (1.0) |
| Kerstjens 2020 | 552 | -1.2 (0.8) | 537 | -1.1 (0.8) |
| Lee 2021 high-dose LAMA | 376 | -0.8 (0.6) | 385 | -0.7 (0.7) |
| Lee 2021 low-dose LAMA | 376 | -0.8 (0.7) | 370 | -0.7 (0.8) |

Study and pooled estimates by inverse variance weighted method for effect size on scale of measurement for ACQ-7

| **Study** | **Difference (95% CI)** | **P** |
| --- | --- | --- |
| Gessner 2020 | -0.09 (-0.22 to 0.03) | 0.15 |
| Kerstjens 2020 | -0.14 (-0.24 to -0.04) | 0.005 |
| Lee 2021 high-dose LAMA | -0.03 (-0.13 to 0.07) | 0.61 |
| Lee 2021 low-dose LAMA | -0.11 (-0.22 to -0.01) | 0.04 |
| Pooled Fixed effect | -0.09 (-0.15 to -0.04) | <0.001 |
| Pooled Random effect | -0.09 (-0.15 to -0.04) | <0.001 |

Heterogeneity and I-squared statistic

| **Chi-square (DF)** | **P** | **I-squared (95% CI)** |
| --- | --- | --- |
| 2.8 (4) | 0.43 | 0 (0.0 to 83.4) |

Publication bias

| **Method** | **Test statistic** |
| --- | --- |
| Correlation coefficient | 0.33  P=0.50 |
